# A Novel Missense Mutation, E1623G, in the Human Factor VIII Gene Associated With Moderate Haemophilia A

**DOI:** 10.5812/ircmj.6727

**Published:** 2014-01-05

**Authors:** Habib Onsori, Mohammad Ali Hosseinpour Feizi, Abbas Ali Hosseinpour Feizi

**Affiliations:** 1 Cell and Molecular Biology Department, Marand Branch, Islamic Azad University, Marand, IR Iran; 2 Biology Department, Tabriz University, Tabriz, IR Iran; 3 Hematology and Oncology Research Center, Tabriz University of Medical Sciences, Tabriz, IR Iran

**Keywords:** Hemophilia A, Mutation, Missense, F8 protein, human

## Abstract

**Introduction:**

Haemophilia A is the most common inherited X-linked recessive bleeding disorder. The severity of the resultant bleeding diathesis depends on the FVIII levels associated with the mutation. Analysis of carrier state can be made indirectly by DNA linkage analysis or directly by identifying the mutation that leads to the disease. The aim of this study was to identification of the causal mutation of the FVIII gene in a haemophilic patient.

**Case Report:**

Our case is a 16-year-old male haemophilia A patient with some symptoms such as recurrent hemarthrosis in left knee. In this study, we used single-stranded conformational polymorphism (SSCP) and conformational sensitive gel electrophoresis (CSGE) methods and direct sequencing to identify the mutation responsible for haemophilia A in our patient.

**Conclusions:**

We reported a novel missense mutation (GAA→GGA), E1623G, in exon 14 of FVIII gene that is associated with moderate haemophilia A. This new mutation was recorded in GenBank (NCBI) with accession number JF916726.1. This study showed that the use of PCR-CSGE and PCR-SSCP may be useful in detecting most of genetic defects such as point mutations of FVIII gene in haemophilic patients.

## 1. Introduction

Haemophilia A is an inherited deficiency of coagulation factor VIII with a frequency of 1 in 10000 males (1, 2). Factor VIII (FVIII) is a glycoprotein cofactor that serves as a critical component in the intrinsic blood coagulation pathway. The gene of FVIII is located at the tip of the long arm of X chromosome. It spans over 180 kb and comprises 26 exons, which encode a polypeptide chain of 2351 amino acids (3). The severity of the resultant bleeding diathesis depends on the FVIII levels witch is associated with the mutation (4). Analysis of carrier state can be made indirectly by DNA linkage analysis or directly by identifying the mutation that leads to the disease. Many mutation screening methods are available but CSGE, a form of heteroduplex analysis, has been used successfully for screening for mutations in haemophilia A and B (5).

## 2. Case Report

A 16-year-old boy was referred to children's hospital of Tabriz, Iran due to recurrent hemarthrosis in left knee in May 30, 2009. Other symptoms such as severe bleeding after circumcise in 6 month old with medical inspections showed probability of haemophilia A. Thus, the patient was subjected to molecular study. The research was done in molecular biology lab at Tabriz University. In order to determine the causal mutation, genomic DNA was extracted from 2.5 mL of EDTA anticoagulated peripheral blood by SDS-proteinase K according to Sambrook et al. (6). Polymerase chain reaction (PCR) of genomic DNA from the normal male subject for all 26 exons with exon-intron boundaries regions were performed in 37 segments with the use of specific primers as reported by Steve Keeney (7). PCR reactions were carried out in 25 μL reaction mixture. The amplified fragments were detected on 1.5% agarose gel by ethidium bromide staining.

**Figure 1.**
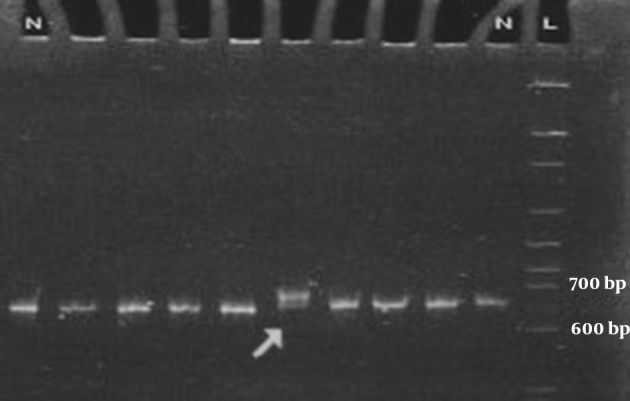
Part of CSGE Gel Showing the Migration Pattern of Exon 14F Amplified Fragments Fragments With Abnormal Migration Patterns are Marked by Arrow. N: Normal Control, L: Ladder.

**Figure 2.**
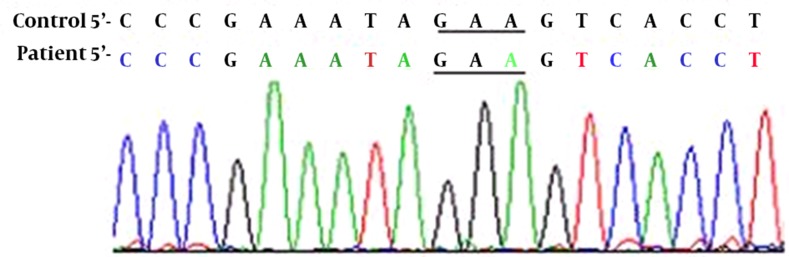
DNA Sequencing Profile of the FVIII Gene (Exon 14F) from the Patient and Normal Controls This Figure Indicates Transition Point Mutation (GAA→GGA) in the Patient DNA Sequence that Replaces a Glutamic acid With a Glycine Residue. Underlining Indicates the Position of Point Mutation.

The PCR-amplified fragments larger than 400bp were subjected to CSGE and the fragments shorter than 400bp were subjected to SSCP analysis. CSGE and SSCP techniques were performed as described before (8). PCR products showing abnormal electrophoretic mobility on SSCP or CSGE gel were sequenced by Kawsar Company and analyzed by sequencing-analysis Chromas Lite 2.01 software. The sequences were compared with the wild type using NCBI BLASTn analyzing software. Among all amplified fragments, only PCR product of exon 14F with 686bp long, displayed unmatched shift in CSGE (Figure 1). A novel point mutation was detected from sequence analysis of exon 14F PCR product (Figure 2). PCR reaction mixture for exon 14 F containing 1 × PCR buffer, 1.5 mM MgCl2, 0.2 mM dNTP, 10 pmoles of each primer (Forward: 5'-TCCCTACGGAAACTAGCAATG-3' and Reverse: 5'-TCACAAGAGCAGAGCAAAGG-3'), 0.5U of Taq DNA polymerase and about 1μg of genomic DNA on a Genius Thermal Cycler. The conditions were as follows: denaturation for 2 min at 95°C, 30 cycles of 30 s at 94°C, annealing for 1 min at 60°C and extension for 30 s at 72°C with a final extension step for for 5 min at 72 °C..

## 3. Discussion

Mutation of the FVIII gene causes deficiency or dysfunction of clotting factor VIII, it is as an essential cofactor of factor X by factor IXa in the presence of Ca2+ and phospholipids (9). FVIII includes a single peptide of 19 amino acids and a mature protein of 2332 amino acids. FVIII is a large multidomain glycoprotein with domain structure A1-A2-B-A3-C1-C2. The large B domain is encoded by a single large exon 14 and has no detectable homology to any other known genes. It is extensively glycosylated on asparagine, serine, and threonine residues (3). Both A and C repeats show conservation of cysteines, and the B region contains most potential N-glycosylation sites. Factor VIII is activated by proteolysis catalyzed by thrombin. The principal activation cleavage sites are on the C-terminal side of arginine residues 372, 740, and 1689. The activation cleavage sites ﬂank the B domain, which is released from factor VIII on activation, leaving a heterotrimer comprising an N-terminal heavy chain and a C-terminal light chain; these are held together by Ca^2+^ (10). Over 50 types nonsense point mutation associated with sever haemophilia A and over 40 types various missense point mutations was reported in exon 14 that most of them occur in codons 1680 and 1689 which cause moderate haemophilia A (11). In this study, we detected a novel missense mutation (GAA→GGA), E1623G, in exon 14 of FVIII gene that causes moderate haemophilia A. This novel missense mutation due to A→G transition at codon 1623 (GAA) of the factor VIII gene replaces a glutamic acid with a glycine residue. In this research, SSCP and CSGE methods were used for mutation detection. They have the advantages of being simple and relatively rapid to perform and do not require the use of radiolabel. However, the techniques require a great deal of skill, both technical and interpretive, to achieve good sensitivity. In conclusion, this study shows that the use of PCR-CSGE, PCR-SSCP and direct sequencing may be useful in detecting most of genetic defects such as point mutations of the FVIII gene in hemophilic patients.
